# The deep sea is a major sink for microplastic debris

**DOI:** 10.1098/rsos.140317

**Published:** 2014-12-17

**Authors:** Lucy C. Woodall, Anna Sanchez-Vidal, Miquel Canals, Gordon L.J. Paterson, Rachel Coppock, Victoria Sleight, Antonio Calafat, Alex D. Rogers, Bhavani E. Narayanaswamy, Richard C. Thompson

**Affiliations:** 1 Department of Life Sciences, The Natural History Museum, Cromwell Road, London SW7 5BD, UK; 2 GRC Geociències Marines, Departament d’ Estratigrafia, Paleontologia i Geociències Marines, Universitat de Barcelona, 08028 Barcelona, Spain; 3 Marine Biology and Ecology Research Centre, School of Marine Science and Engineering, Plymouth University, Plymouth, Devon PL4 8 AA, UK; 4 Department of Zoology, University of Oxford, Tinbergen Building, South Parks Road, Oxford OX1 3PS, UK; 5 The Scottish Association for Marine Science, Scottish Marine Institute, Oban, Argyll PA37 1QA, UK

**Keywords:** marine, litter, plastic, fibres, seabed, microplastic

## Abstract

Marine debris, mostly consisting of plastic, is a global problem, negatively impacting wildlife, tourism and shipping. However, despite the durability of plastic, and the exponential increase in its production, monitoring data show limited evidence of concomitant increasing concentrations in marine habitats. There appears to be a considerable proportion of the manufactured plastic that is unaccounted for in surveys tracking the fate of environmental plastics. Even the discovery of widespread accumulation of microscopic fragments (microplastics) in oceanic gyres and shallow water sediments is unable to explain the missing fraction. Here, we show that deep-sea sediments are a likely sink for microplastics. Microplastic, in the form of fibres, was up to four orders of magnitude more abundant (per unit volume) in deep-sea sediments from the Atlantic Ocean, Mediterranean Sea and Indian Ocean than in contaminated sea-surface waters. Our results show evidence for a large and hitherto unknown repository of microplastics. The dominance of microfibres points to a previously underreported and unsampled plastic fraction. Given the vastness of the deep sea and the prevalence of microplastics at all sites we investigated, the deep-sea floor appears to provide an answer to the question—*where is all the plastic?*

## 2. Background

Plastics are extremely durable synthetic polymers, yet more than 30% are made into disposable items such as packaging, which are typically discarded within a year of manufacture [1]. The associated throw-away culture has led to an escalating plastic waste management problem, and widespread accumulation of plastic debris in the natural environment. Debris is now present on shorelines and at the sea surface from pole to pole [1,2]. It has major environmental impacts and is recognized as one of the key challenges of our century [1–3]. However, despite extensive environmental monitoring, there is little evidence of the expected increasing abundance of plastic debris in natural habitats. Only two studies [4,5] report an increase over time. Both these papers focused on microplastics, which have not typically been included in routine monitoring, and are likely to represent a largely undocumented accumulation of plastic debris. Yet, even for microplastic pollution, temporal trends are unresolved in the majority of datasets [6]. In addition, a recent study [7] suggested that surface water plastic accumulation was tens of thousands of tonnes less than expected, and acknowledged that resolving the fate of the missing plastic is a fundamental issue [8].

Plastics can be denser (e.g. acrylic) or lighter (e.g. polypropylene) than seawater. Those that are buoyant float when first entering the sea, so historically attention has focused on the accumulation on shorelines and at the sea surface [9]. However, because of fouling by organisms and adherence of particles, positively buoyant plastics can, over a timescale of weeks to months, become negatively buoyant and sink [10]. Some studies have shown the accumulation of large plastic items in the deep sea [11,12], and one has reported the presence of microplastic fragments at low densities [13].

Here, we present results from a global analysis of deep-sea sediment collected by two independent research teams during seven research cruises between September 2001 and August 2012, in the Mediterranean Sea, SW Indian Ocean and NE Atlantic Ocean (spanning subtropical to subpolar waters). The purpose of this study was to quantify the abundance and extent of microplastic contamination at a range of depths and locations in the deep sea.

## 3. Methods

### 3.1 Sample collection

**Figure 1.**
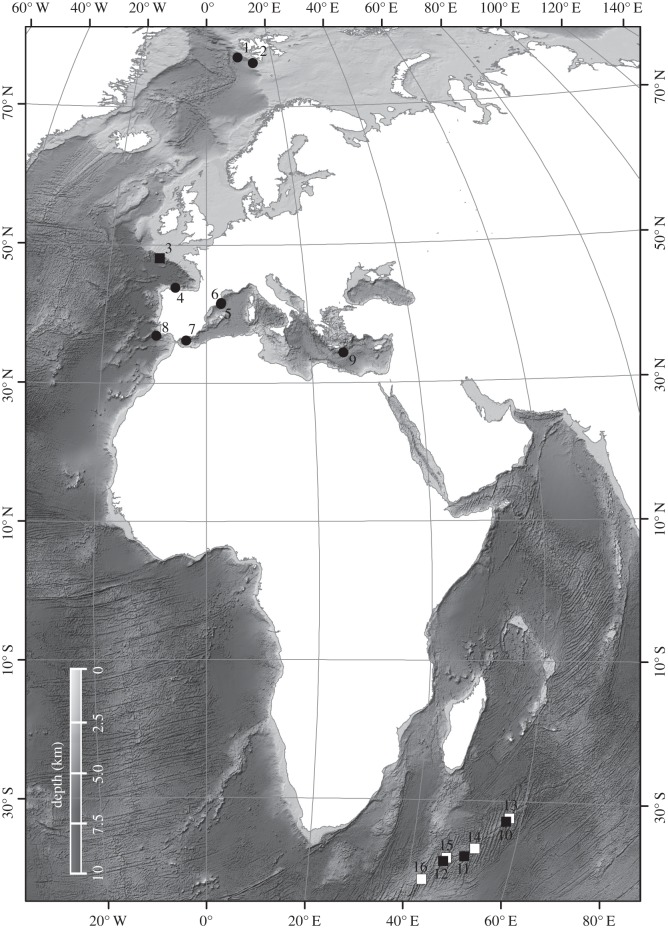
Locations of sampling sites of bottom sediment and deep-water coral where content of microplastics was investigated. Sample depth ranged down to 3500 m, for details see table 1. Sediment was collected by the University of Barcelona (circles) and the Natural History Museum (filled squares), and deep-water corals were collected by the Natural History Museum (open squares). Bathymetry corresponds to ETOPO1 Global Relief Model.

Deep-sea sediment cores from environments such as submarine canyons, continental slopes, basins and seamounts were collected independently by the University of Barcelona and the Natural History Museum, London. In addition, coral specimens were sampled on seamounts (figure 1 and table 1). Sampling depth ranged down to 3500 m, but most sites were at around 1000 m and were at least 9 km horizontal and 200 m vertical distance from each other. In total, 12 quantitative sediment cores and four qualitative coral samples were collected.

**Table 1.** Details of sampling location and quantity of microplastics found in the North Atlantic Ocean, Mediterranean Sea and SW Indian Ocean. UB, University of Barcelona; NHM, Natural History Museum, London; P, microplastic fibres present.

sample no.	sampler	location	depth (m)	province	microplastic abundance
1^a^	UB	subpolar N Atlantic	2000	open slope	15
2^a^	UB	subpolar N Atlantic	1000	open slope	10
3^a^	NHM	NE Atlantic	1400	canyon	6
4^a^	UB	NE Atlantic	2000	canyon	40
5^a^	UB	Mediterranean	300	canyon	35
6^a^	UB	Mediterranean	1300	canyon	10
7^a^	UB	Mediterranean	900	open slope	10
8^a^	UB	NE Atlantic	2200	open slope	10
9^a^	UB	Mediterranean	3500	basin	15
10^a^	NHM	SW Indian	900	seamount	3.5
11^a^	NHM	SW Indian	1000	seamount	4
12^a^	NHM	SW Indian	900	seamount	1.4
13^b^	NHM	SW Indian	800	seamount	P
14^b^	NHM	SW Indian	700	seamount	P
15^b^	NHM	SW Indian	800	seamount	P
16^b^	NHM	SW Indian	500	seamount	P

^a^Quantified as plastic fibres per 50 ml sediment.

^b^Unquantified and from coral specimens.

Sediment cores 1, 2 and 4–9 were collected by the University of Barcelona on board the research vessels *Jan Mayen* (Norway), *Garcia del Cid* (Spain), *Sarmiento de Gamboa* (Spain) *Hespérides*(Spain) and *Aegaeo* (Greece). Ten centimetre diameter cores were obtained from megacorers or boxcores that were subsequently subsampled. The top 1 cm of the sediment cores was sliced on board the ship and then frozen at −20^°^C. Upon arrival in the laboratory, the sediment was freeze-dried and stored at room temperature in sealed containers for a maximum of 12 years before sending to Plymouth University for extraction of microplastics.

Sediment cores 3 and 10–12 and corals 13–16 were sampled by the Natural History Museum, London. Sampling was performed during research cruises JC66 and JC76T aboard the *R.R.S. James Cook* (UK). Samples were collected by remotely operated vehicle (ROV) *Kiel6000* (Germany) or*ISIS* (UK), using push-cores or a megacorer for sediment samples or ROV manipulator arms for the corals. Cores were of 5.7, 7.4 or 10 cm internal diameter, respectively. Once recovered on board the ship, cores were immediately sliced at 2 cm and 5 cm using a metal plate. Sediment horizons were preserved in either DESS [14] or 4% formalin in sealed containers for a maximum of a year. All preservation and processing fluids were filtered through a 32 μm sieve prior to adding them to the sediment. Filtered (5 μm) fresh water and seawater were used to wash equipment, and water from both fresh and salt water supplies was sampled for 1 h daily through a 32 μm sieve and screened for the presence of microplastic fibres, but none was seen.

### 3.2 Sample processing

All sample processing (extraction and picking) was conducted in a clean laboratory, where extreme care was taken to avoid any contamination. Checks for contamination during processing were made by exposing damp filter paper to the air in the laboratory, whenever samples were open to the laboratory environment. As an additional precaution, those handling the samples wore only natural fibre clothing, and were protected with 100% cotton laboratory coats and headwear, and latex gloves, for all laboratory processing and during the JC76T research cruise.

Microplastics were extracted from the sediment using two methodologies. For samples 1, 2 and 4–9, extraction was done by Plymouth University (PU) using a concentrated NaCl solution and filtering with three sequential extractions [5]. The PU method employs supernatant filtering through a Whatman GF/A filter. For samples 3 and 10–12, particle extraction was conducted at the Natural History Museum (NHM) using an adapted Ludox-TM 40 extraction method [15] employing eight centrifuge cycles and a 32 μm sieve to separate the microplastics from the sand grains. The substances chosen to isolate the fibres (NaCl or Ludox-TM 40) had similar specific gravities, namely 1.2 and 1.16, for PU and NHM, respectively. We are therefore confident that the same fractions and types of microplastics were isolated.

Using an entomological pin, microplastic fibres from coral specimens 13–16 were removed under a binocular microscope and placed into clean vials containing Millipore water. The fibres were not extracted quantitatively. The corals were of different sizes, and not all fibres present on the corals were removed, therefore just the presence or absence of microplastic accumulation on living coral was recorded.

All sediment samples were examined under a binocular microscope, and any objects that were of unnatural appearance based on shape and colour (potential microplastics) were transferred to sealed containers and subsequently identified [5] by spectrometry.

### 3.3 Microplastic analyses

A Bruker IFS66 Fourier transform-infrared (FT-IR) spectrometer with a mercury cadmium telluride detector operating in the 4000–600 cm^−1^ wavenumber range was used for object identification. A Specac DC2 Diamond compression cell (2 mm in diameter) was used to allow transmission of the IR beam to the detector. Bruker’s Opus 5.5 spectroscopy software was used for measurement, processing and evaluation of the IR spectra. Matches with quality index greater than or equal to 0.7 were accepted. Matches with quality index less than 0.7, but greater than or equal to 0.6 were individually inspected and interpreted based on the closeness of their absorption frequencies to those of chemical bonds in the known polymers. Matches with quality index less than 0.6 were rejected. We added the spectra of potential contaminants, such as those from waterproofs and laboratory gloves used during sample collection and processing, to the Plymouth University FT-IR library in order to eventually eliminate any contamination from the data. No matches with these materials were found in any of the samples. In addition, we checked all residue sediment for the larger plastic particles described in Van Cauwenberghe *et al*. [13]. None were seen. Protocols were implemented to prevent plastic contamination from the processing environment and controls to monitor air and water supplies were taken during all processing phases.

There has only been one previous report of microplastics in the deep sea, which documented very low abundance [13]. The previous study employed different separation methods to those used here and critically did not enumerate microplastic fibres, which are the most numerous type of particle present in shallow water habitats and in some biota [5]. We ran a preliminary trial to compare methods used by both studies, by splitting some of our deep-sea sediment samples and analysing them using both approaches. We found that our approach and our recording of microfibres yielded substantially greater abundance of microplastic particles, whereas the other method [13] underestimated the microplastic concentration (electronic supplementary material).

## 4. Results

**Figure 2.**
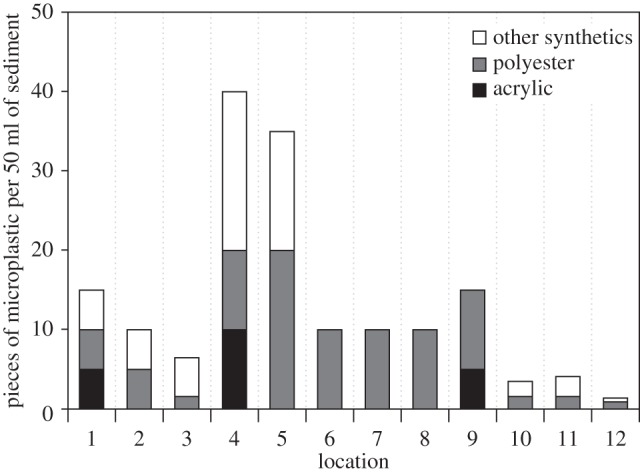
The quantity and type of plastic fibres found in 50 ml of sediment sampled from the North Atlantic Ocean, Mediterranean Sea and SW Indian Ocean.

Identification by FT-IR spectroscopy confirmed that microplastics were abundant in all 12 sediment samples and all coral samples. The microplastics were all fibrous in shape, were commonly 2–3 mm in length and less than 0.1 mm in diameter (electronic supplementary material, figure S1). Plastic microfibre abundance in the sediments ranged from 1.4 to 40 pieces per 50 ml (mean±s.e.: 13.4±3.5; figure 2), and samples from four locations in the Indian Ocean showed that microplastics had also accumulated on the surface of octocorals. The microfibres were mostly blue, black, green or red, although vibrant colours such as pink, purple and turquoise were also seen. Rayon, which is a man-made non-plastic polymer, was detected in all the samples (electronic supplementary material, figure S2*a*). It contributed to 56.9% of the total number of fibres seen and was more than twice as abundant as polyester (electronic supplementary material, figure S2*b*). Of the remaining fibres, polyester was the most prevalent (53.4%), followed by other plastics (34.1%), which included polyamides and acetate, then acrylic (12.4%) (figure 2).

## 5. Discussion

Because of the lack of replicate samples, small sample size and differences in methodology, no attempt can be made to statistically compare microplastic abundance and composition between samples and make conclusions on patterns observed. However, we can and do compare the mean abundance reported here with that of other studies, highlighting the ubiquity of the microplastic presence and emphasizing that the microplastics reported were all fibres.

The prevalence of plastic microfibres in all sediment cores and on all coral colonies examined suggests this contaminant is ubiquitous in the deep sea. Furthermore, the wide variety of polymer types detected reveals that the accumulation and deposition of microfibres in the deep sea is complex and that they arise from a variety of domestic and industrial sources.

The microplastics, in the form of microfibres, were found in the deep sea in similar abundance to those reported in intertidal or shallow subtidal sediments (respectively, mean±s.e.: 3.3±2.7 and 6.4±2.7 per 50 ml; table 2). While different methods are used to sample surface waters and sediments, a qualitative comparison indicated four orders of magnitude greater abundance per unit volume in deep-sea sediments compared with heavily contaminated surface water gyres (1.1×10^−4^ per 50 ml; table 2). Our data show that, if extrapolated and using the most conservative estimates, 4 billion fibres per km^2^ would be present in Indian Ocean seamount sediment. It is notable that fibres represented the largest proportion of microplastics seen in other studies based on sediment analyses, whereas sea-surface studies most often report larger fragments. At present, it is not possible to determine whether this is a true reflection of the relative abundance of different microplastic fractions in different substrata or if it is an artefact of differences in sampling methodology [23].

**Table 2.** Concentrations of microplastics previously reported in shallow water marine sediments and surface waters worldwide. This present study reports 13.4 pieces per 50 ml of sediment.

region	pieces per 50 ml	reference
sediment		
subtidal, UK	6	Thompson *et al*. [5]
estuary, UK	4	
beach, UK	0.5	
beach, Chagos Arch., Indian Ocean	4.5	Readman *et al.* [16]
beach, worldwide	0.4–6.2	Browne *et al.* [17]
subtidal, UK	0.2–1	
average	3.7^a^	
surface water		
NE USA coast	0.0000675^b^	Carpenter & Smith [18]
North Pacific Gyre	0.0001115^b^	Moore *et al.* [19]
S California coast	0.0003625^b^	Moore *et al.* [20]
NE Pacific Ocean coast	0.00000485^b^	Doyle *et al.* [21]
NW Mediterranean Sea	0.0000058^b^	Collignon *et al.* [22]
average	0.00011043	

^a^Worldwide shore sites cited by Browne *et al.* [17] not included.

^b^Extrapolated values.

Polyester, which forms the largest proportion of plastic microfibres in this study, is often the most common polymer detected in some other microplastics studies [17,24–26]. However, a study which sampled the water column concluded that other plastics such as polypropylene and polyethylene are most abundant [27]. Most of the polymer types recorded in this study are negatively buoyant, and will therefore eventually sink. This may account for the difference in microfibre composition between this benthic study and the water column study. The plastic polymers found in this study are used in a wide range of domestic and industrial applications, including packaging, textiles and electronics, which indicates diverse sources. However, the precise origin of individual microplastic debris cannot currently be established. Rayon is not a plastic, but we include it in our results, because it is a man-made semi-synthetic material and widely reported as present in the marine environment. It is used in cigarette filters, personal hygiene products and clothing, and is introduced to the marine environment through sewage, including from the washing of clothes. It has been reported in fish (57.8% of synthetic particles ingested) [24] and in ice cores (54%) [25], in similar proportions to those reported here.

There is precedent for the transport of natural particulates from shallow water to the deep sea, so similar processes may be involved with microplastics. Some negatively buoyant particles will sink; however, because of surface tension and oceanographic currents, most plastics are initially held at the sea surface. Colonization by organisms, adherence to phytoplankton and the aggregation with organic debris and small particles in the form of marine snow will eventually enhance settling. In addition, a number of oceanographic processes could aid in the transfer of microplastics to depth. These processes include dense shelf water cascading [28,29], severe coastal storms [30], offshore convection [31,32] and saline subduction [33]. All these induce vertical and horizontal transfers of large volumes of particle loaded waters, including a full spectrum of grain sizes (from sand to clay), as well as litter and contaminants, from shallow ocean layers and coastal regions to deeper ones [11–13], with submarine canyons acting as preferential conduits [34]. Submarine topographic features may also enhance downwelling flows and increase the retention of microplastics at particular locations such as Taylor columns over seamounts [35]. Microplastic fragments are also more likely than larger items to be influenced by advection and, more generally, circulation patterns at all ocean levels, because of their small size. Hence, ocean dynamics can explain the accumulation of plastics in the deep sea.

## 6. Conclusion

Our results show for the first time, to the best of our knowledge, that substantial quantities of microplastic debris have accumulated in the deep sea. Given the extent of this habitat (more than 300 million km^2^) [36], it therefore seems likely that the deep sea is a sink for this debris. Thus, we have started to elucidate the location of the missing ocean plastic [7]. Plastic accumulation is a global concern because of its effects on marine organisms. The discovery of previously under-reported microplastics suggests that there may be even greater plastic accumulation than was previously suspected. In contrast with studies on the effects of large plastic debris, which mostly document entanglement and ingestion [37], there are few studies examining the biological effects of microplastics. A range of organisms are known to ingest microplastics, and there is concern this could result in physical and/or toxicological harm [38,39]. The extent to which microplastics could have harmful effects will most likely be influenced by their relative abundance. The discovery of substantial quantities in deep-sea sediments is of considerable relevance to our understanding of the potential of these particles to cause harm in the marine environment.

To date, our understanding regarding the dynamics of transport, accumulation and associated spatial distribution has been extremely limited, and the data presented here, together with that of Van Cauwenberghe *et al.* [13], provide the first evidence of global sinks for microplastic debris, a theory previously suggested for larger plastic debris items [40]. It is now crucial to establish consistent methodologies to allow robust temporal and spatial comparisons, to address how abundance and composition vary with depth, location, topography and habitat, and apply these data to the already complex oceanographic transport models available for some oceans, which have successfully been used to predict surface plastic accumulation [6]. In addition, the elucidation of the physical and toxicological effects of microplastics is also required. In summary, further data collection is required to properly establish the impact of microplastic particles on deep-sea communities and related ecosystem services.

## Supplementary Material

Table S1: A comparison of methods using during this study at University of Barcelona (UB), Plymouth University (PU) and Natural History Museum (NHM), and used by Van Cauwenberghe et al. (2013). The table is followed by a short discussion about the methodological differences. Table S2: Raw data showing sample extraction method, volume of sediment processed and the number and type of fibers observed. Table S3: Raw data showing number and type of fibres removed from coral samples 13–16. Data is qualitative and given to show the range of polymer types encountered.

## Supplementary Material

Fig S1. Example of microplastic fibre detected in deep-sea sediment.

## Supplementary Material

Fig S2. The quantity and type of plastic and rayon fibres found in 50 ml of sediment (a) by sample, (b) total proportion of each microfibre type. The following are all in the same file

## 7. Data Accessibility

The datasets supporting this article have been uploaded as the electronic supplementary material.
