# Supplementary material for: The deep sea is a major sink for microplastic debris
Source: R Soc Open Sci. 2014 Dec 17;1(4):140317. doi: 10.1098/rsos.140317 (PMC4448771; doi:10.1098/rsos.140317)
Supplement: Table S1: A comparison of methods using during this study at University of Barcelona (UB), Plymouth University (PU) and Natural History Museum (NHM), and used by Van Cauwenberghe et al. (2013). The table is followed by a short discussion about the methodological differences. Table S2: Raw data showi [file rsos140317supp1.docx]

**Table S1: A comparison of methods used during this study at University of Barcelona (UB), Plymouth University (PU) and Natural History Museum (NHM), and used by Van Cauwenberghe *et al.* (2013).**

|  | Institution/ Study | | |
| --- | --- | --- | --- |
| Process | UB/PU | NHM | Van Cauwenberghe *et al.* (2013) |
| Sample Collection |  |  |  |
| Sediment core depth | Top 1 cm | Top 2 and 5 cm | Top 1 cm |
| Core diameter | 10 cm | 5.7, 7.4, 10 cm | 5.6 cm |
| Preservation method | -20ºC | DESS | Unknown |
| Subsequent processing | Freeze dried | NONE | Unknown |
| Sample Extraction |  |  |  |
| Extraction medium | NaCl | Ludox | NaI |
| Specific gravity | 1.2 | 1.16 | 1.6 |
| Settling method | Gravity | Centrifuge | Centrifuge |
| Sequential repeats | 5 | 8 | Unknown |
| Filter/ Sieve size | Whatman GF/A filter | 32 µm sieve | 1mm then 35 µm sieves |

Methods comparison

There were differences between the collection and extraction procedures of the samples 1, 2, 4-9 and 3, 10-12 which were processed by UB/PU and NHM respectively. The main differences during the collection stage of the samples was depth of sediment preserved and the method of preservation. The NHM core samples included deeper sediment. Due to sediment layer mixing via bioturbation and current surface effects there is unlikely to be major effect of the sediment depth, however there could a greater abundance of microplastics in the top few mm of the cores, which would result in lower abundance of microplastics in NHM samples. Further work would be needed to establish the depth profile of microplastics in sediments. The preservation methods of freezing and DESS would not influence microplastic abundance as the DESS was filtered prior to being added to the sediment sample.

Sample processing differed with different fluids being used for extraction. However both the liquids had approximately the same specific gravity and therefore would have extracted particles with similar densities. The NHM method utilised a centrifuge and samples underwent more cycles of processing than the UB/PU samples. The efficiency of these methods was not compared, but observations showed that the NHM method had removed more than 90% of particles after 5 cycles (unpublished data), therefore the additional cycles may have had little effect. Furthermore the filter used for the UB/PU samples was of smaller pore size than the sieve used for the NHM samples, thus the NHM samples may have not recovered some of the smallest plastics. Further work would be needed to make a formal comparison of these methods.

Comparing the methods used in this study and those employed by Van Cauwenberghe *et al.* showed that the Van Cauwenberghe *et al* method extracted fewer fibres than that used for the UB/PU samples (Table S2). In addition the Van Cauwenberghe *et al.* study specifically excluded the fibre proportion of the microplastics (Van Cauwenberghe, pers comm.). All these aspects highlight the potential impact of different methods and processes and underscore the importance of developing standard methods which would allow for quantitative comparison across studies.

| Sample location | Extraction method | Total volume of sediment processed (ml) | Acrylic | Polyester | Rayon | PBT | Nylon | Acetate | Other synthetic/  Unknown | Total |
| --- | --- | --- | --- | --- | --- | --- | --- | --- | --- | --- |
| 1 | Thompson *et al.* (2004) [5] | 10 | 1 | 1 | 1 | 0 | 0 | 0 | 1 | 4 |
| 2 | Thompson *et al.* (2004) [5] | 10 | 0 | 1 | 1 | 0 | 0 | 0 | 1 | 3 |
| 4 | Thompson *et al.* (2004) [5] | 10 | 2 | 2 | 10 | 0 | 1 | 0 | 3 | 18 |
| 5 | Thompson *et al.* (2004) [5] | 10 | 0 | 4 | 5 | 0 | 0 | 1 | 2 | 12 |
| 6 | Thompson *et al.* (2004) [5] | 10 | 0 | 2 | 3 | 0 | 0 | 0 | 0 | 5 |
| 7 | Thompson *et al.* (2004) [5] | 10 | 0 | 2 | 8 | 0 | 0 | 0 | 0 | 10 |
| 8 | Thompson *et al.* (2004) [5] | 10 | 0 | 2 | 3 | 0 | 0 | 0 | 0 | 5 |
| 9 | Thompson *et al.* (2004) [5] | 10 | 1 | 2 | 3 | 0 | 0 | 0 | 0 | 6 |
|  |  |  |  |  |  |  |  |  |  |  |
| 2 | Van Cauwenberghe *et al.* (2013) [12] | 10 | 0 | 0 | 0 | 0 | 0 | 0 | 1 | 1 |
| 6 | Van Cauwenberghe *et al.* (2013) [12] | 10 | 0 | 0 | 0 | 0 | 0 | 0 | 0 | 0 |
| 7 | Van Cauwenberghe *et al.* (2013) [12] | 10 | 0 | 0 | 2 | 0 | 0 | 0 | 0 | 2 |
| 8 | Van Cauwenberghe *et al.* (2013) [12] | 10 | 0 | 0 | 3 | 0 | 0 | 0 | 0 | 3 |
|  |  |  |  |  |  |  |  |  |  |  |
| 3 | adapted from Griffiths *et al.* (1990) [14] | 128 | 0 | 2 | 19 | 0 | 1 | 0 | 4 | 26 |
| 10 | adapted from Griffiths *et al.* (1990) [14] | 215 | 0 | 7 | 20 | 2 | 0 | 0 | 6 | 35 |
| 11 | adapted from Griffiths *et al.* (1990) [14] | 215 | 0 | 7 | 24 | 1 | 0 | 0 | 8 | 40 |
| 12 | adapted from Griffiths *et al.* (1990) [14] | 215 | 0 | 4 | 12 | 0 | 0 | 0 | 2 | 18 |

**Table S2: Raw data showing sample extraction method, volume of sediment processed and the number and type of fibres observed.**

**Table S3: Raw data showing number and type of fibres removed from coral samples 13-16. Data is qualitative and given to show the range of polymer types encountered.**

| Sample ID | Polyester | Rayon | Nylon | Other synthetic/  Unknown | Total |
| --- | --- | --- | --- | --- | --- |
| 13 | 6 | 1 | 0 | 0 | 7 |
| 14 | 2 | 10 | 1 | 0 | 13 |
| 15 | 3 | 7 | 0 | 2 | 12 |
| 16 | 1 | 5 | 0 | 0 | 6 |
