# Supplementary figures and images for: The deep sea is a major sink for microplastic debris
Source: R Soc Open Sci. 2014 Dec 17;1(4):140317. doi: 10.1098/rsos.140317 (PMC4448771; doi:10.1098/rsos.140317)

Figure S1. Example of microplastic fibre detected in deep-sea sediment.

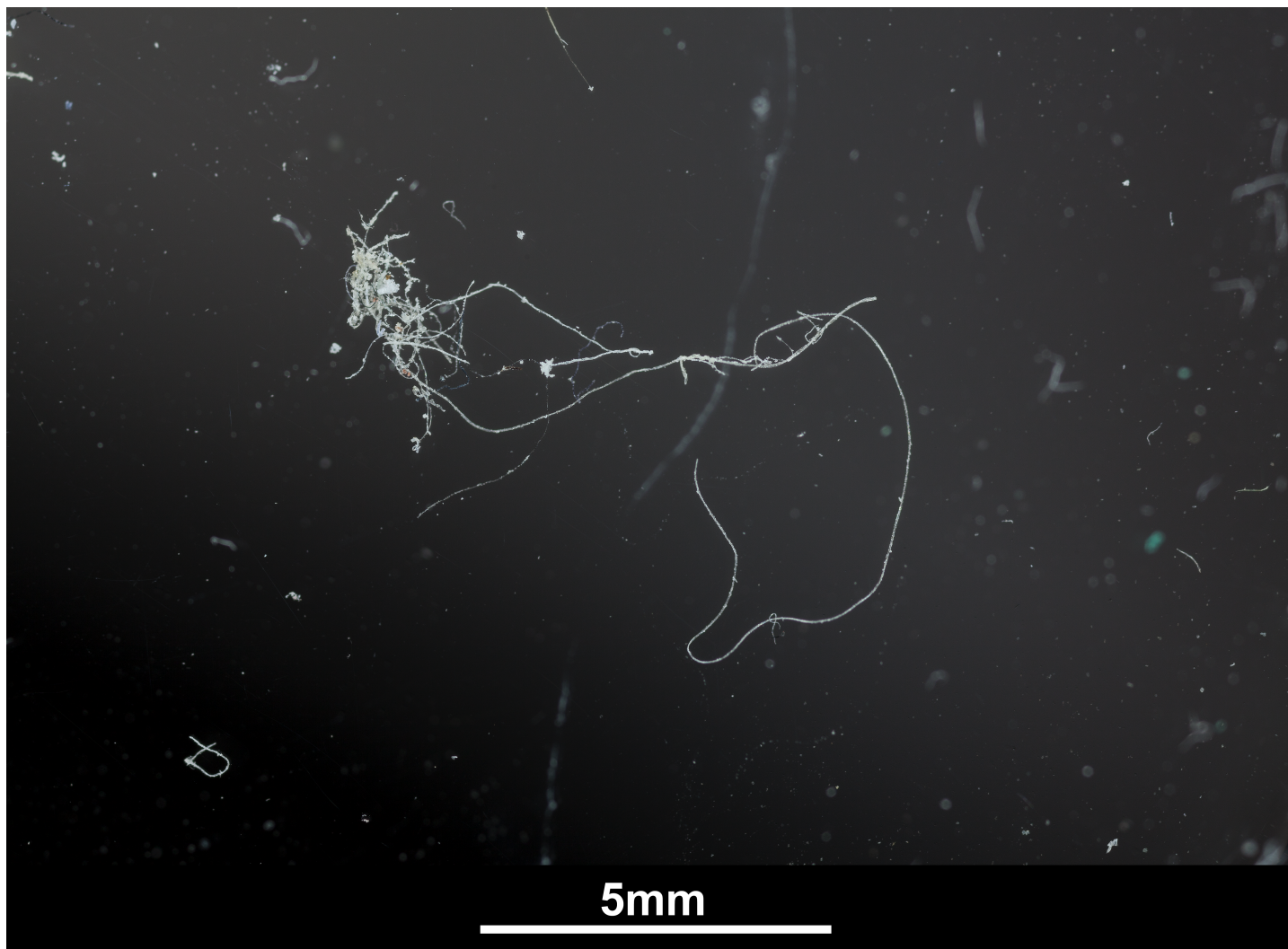

Supplement: Fig S1. Example of microplastic fibre detected in deep-sea sediment. [file rsos140317supp2.pdf]
