# Supplementary material for: The deep sea is a major sink for microplastic debris
Source: R Soc Open Sci. 2014 Dec 17;1(4):140317. doi: 10.1098/rsos.140317 (PMC4448771; doi:10.1098/rsos.140317)

Figure S2. The quantity and type of plastic and rayon fibres found in 50 ml of sediment (a) by sample, (b) total proportion of each microfibre type.

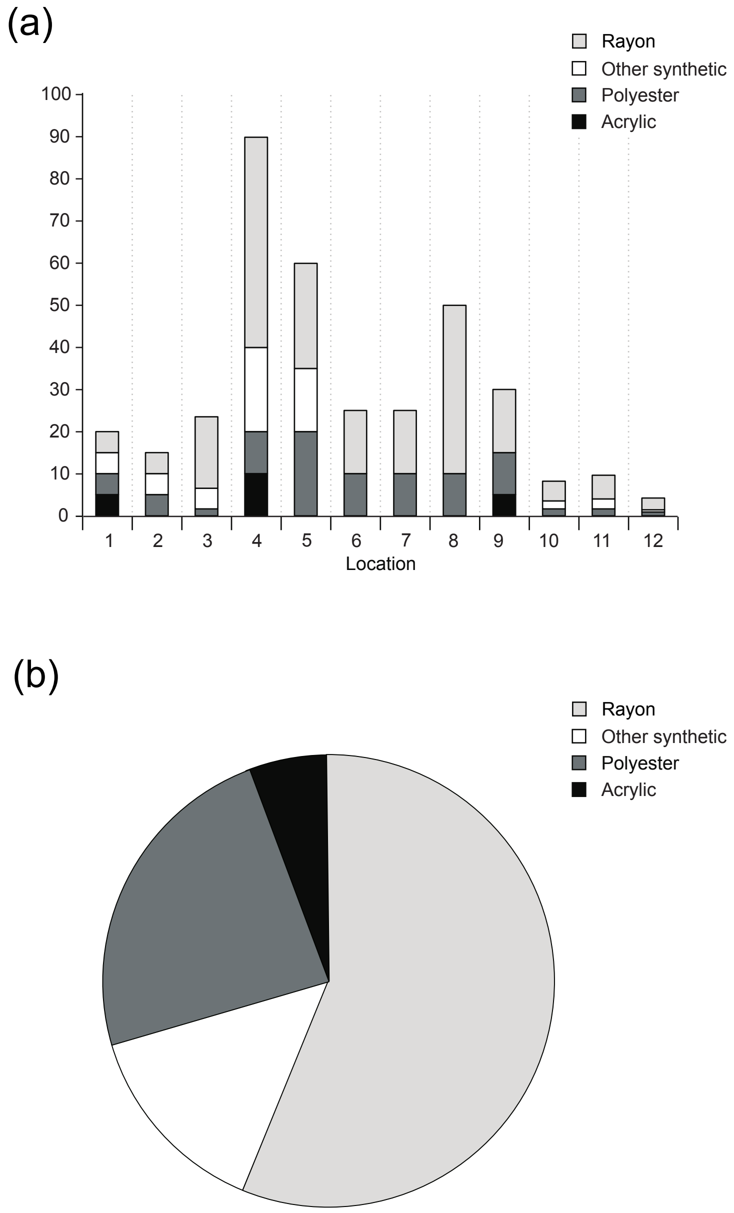

Supplement: Fig S2. The quantity and type of plastic and rayon fibres found in 50 ml of sediment (a) by sample, (b) total proportion of each microfibre type. The following are all in the same file [file rsos140317supp3.pdf]
